# A secure key dependent dynamic substitution method for symmetric cryptosystems

**DOI:** 10.7717/peerj-cs.587

**Published:** 2021-07-19

**Authors:** Aisha Ejaz, Ijaz Ali Shoukat, Umer Iqbal, Abdul Rauf, Afshan Kanwal

**Affiliations:** 1 Riphah College of Computing, Riphah International University Faisalabad Campus, Faisalabad, Pakistan; 2 Department of Mathematics, COMSATS Institute of Information Technology, Sahiwal Campus, Sahiwal, Pakistan

**Keywords:** Dynamic substitution method, Cryptography, Symmetric cryptosystems

## Abstract

The biggest challenge for symmetric cryptosystems is to replace their static substitution with dynamic substitution, because static substitution S-boxes make the symmetric block ciphers more vulnerable to attacks. Previous well-known dynamic key-dependent S-boxes are lacking in dynamicity and do not provide optimal security for symmetric block ciphers. Therefore, this research aims to contribute an effective and secure method for designing key-dependent dynamic S-box with dynamic permutations to make the symmetric block ciphers optimally secure. The proposed S-box method has been experimentally evaluated through several measures such as bit independence criteria, non-linearity, hamming distance, balanced output, strict avalanche criteria including differential and linear approximation probabilities. Moreover, the randomness properties of proposed method have also been evaluated through several standard statistical tests as recommended by the National Institute of Standards and Technology (NIST). Thus, the results show that the proposed method, not only retains effective randomness properties but it also contains, good avalanche effect (up to 62.32%) which is significantly improved than others. Therefore, the proposed substitution method is highly sensitive to the secret key because, only a single bit change in key generates an entirely new S-box with all 256 values at different positions. Thus, the overall evaluation shows that the proposed substitution method is optimally secure and outperforming as compared to the existing S-box techniques. In future, the proposed method can be extended for different key sizes (192–256 bits) or even more.

## Introduction

During the past two decades, the designing of a key-dependent S-box method with randomized properties has become the utmost need of current and future cryptosystems. Although current cryptosystems offer various S-box solutions (Ahmad et al., 2015; Kumar, Munjal & Sharma, 2011; Sasi, Dixon & Wilson, 2014; Ebrahim, Khan & Khalid, 2014), all these S-box solutions are not optimally effective in dynamic and randomized properties at the time of generating dynamic S-box matrix with all 256 values at different positions. As the technology is evolving day by day (Niksaz, 2015; Tewari & Verma, 2016; Wang & Liu, 2015), people are looking for new and novel ways for data communication over the networks (Jones & Katzis, 2018) with optimal data security. During the communication and transmission of data, it is highly probable that adversaries or malicious users can have potential access to the sensitive information (Thamilarasu & Odesile, 2017) and, as a result, the security of any network or any communication system may be at risk (Anees, Siddiqui & Ahmed, 2014; Peng & Jin, 2013). However, there is no risk that is ever acceptable over the security of secret data contents (Afsana et al., 2018; Shen et al., 2015). In today’s smart life, there is a race between “cryptography” and “cryptanalysis” which results in continuous improvements in cryptography and cryptanalysis (Dooley, 2013; Morain, 1997; Singh, 2003). New cryptanalysis techniques with modern attacks (Ayushi, 2010) such as differential and linear attacks (Chabaud & Vaudenay, 1995; Biryukov et al., 2009; Piper & Murphy, 2002; Bahrak & Aref, 2007), attacks for key recovery (Biryukov et al., 2009) and shortcut attacks (Health, 2016; Daemen & Rijmen, 2002; Rahman, Miah & Azad, 2014) are creating the need of dynamic natured secure cryptosystems with randomized substitution methods. It is widely believed that any cryptosystem having good dynamic and randomness properties can easily resist these kind of modern attacks.

Critical analysis of well-known symmetric block ciphers such as Advanced Encryption Standard (AES) (Sanchez-Avila & Sanchez-Reillo, 2001; Goel, Mehla & Karnal, 2014; Daemen & Rijmen, 2000), Data Encryption Standard (DES) (Sanchez-Avila & Sanchez-Reillo, 2001), Triple-DES (Goel, Mehla & Karnal, 2014) and other well-known symmetric cryptosystems clearly show that the substitution is the basic source of non-linearity in block ciphers (Zhang, 2018; Kazlauskas, Vaicekauskas & Smaliukas, 2015; Gangadari & Ahamed, 2015). Nonlinear substitution is the basic strength of symmetric cryptosystems (Easttom, 2018; Kazlauskas, Vaicekauskas & Smaliukas, 2015). S-boxes are used as look up matrix to alter plaintext into certain secret substitutes in symmetric cryptosystems (Du et al., 2016; Gangadari & Ahamed, 2015) to establish confusion by mapping m-bits into n-bits binary digits (Patil et al., 2016; Picek et al., 2014; Dara & Manochehri, 2013; Agarwal, Singh & Kilicman, 2018; Katiyar, Jeyanthi & Roperties, 2016). Thus, for creating optimal confusion and diffusion, the permutations and substitutions are significant tools for modern cryptosystems (Agarwal, Singh & Kilicman, 2018; Hussain et al., 2010). However, most of existing block ciphers are based on fixed and static natured s-boxes which are the basic design level flaw of symmetric block ciphers (Dharbhashayanam, Chari & Dharbhashayanam, 2018; Maram & Gnanasekar, 2016). Therefore, predefined (static) substitution is the most prominent weakness associated with symmetric cryptosystems (Stoianov & Altimirski, 2012; Xu, Liu & Wu, 2018; Fink et al., 2017) because fixed and predefined diffusion and confusion properties lead to insecure ciphers (D’souza & Panchal, 2018; Xu, Liu & Wu, 2018). Although permutation has its own impact, substitution is the basic root of security in encryption algorithms (Mar & Latt, 2008).

Moreover, predefined (fixed) S-boxes are not dependent on the secret key due to which these type of static S-boxes provide easy trapdoors for the attackers to launch algebraic attacks (Fink et al., 2017; Manjula & Mohan, 2017; Suana, 2018). Therefore, the upcoming challenge for the current symmetric cryptosystems is to evolve their predefined S-box structure with dynamic tactics (Katiyar, Jeyanthi & Roperties, 2016; Dara & Manochehri, 2014; Shoukat et al., 2020a) for the purpose of resisting differential and linear attacks as discussed in Ara, Shah & Prabhakar (2018) and Luma, Hilal & Ekhlas (2015). The cryptographic strength of ciphers can be increased by generating S-boxes dynamically, as stated in Hosseinkhani & Haj Seyyed Javadi (2012) and Shoukat et al. (2020b) because the encryption key is the only secret and changing parameter during encryption process (Agarwal, Singh & Kilicman, 2018; Ahmed & Elkamchouchi, 2013).

Several S-boxes have been designed by researchers (Zahid, Arshad & Ahmad, 2019; Ahmad, Doja & Beg, 2018; Hussain et al., 2018; Das, Zaman & Ghosh, 2013) and numerous new techniques have been proposed for construction of strong S-boxes. Meanwhile, the existing S-box solutions are either static (fixed) in nature or their dynamic structure is lacked in establishing of dynamic s-box belongings with entirely different s-box values (all 256 values) at different positions due which they are quiet vulnerable to modern attacks (Devi, Sharma & Rangra, 2015). Thus, there is need for a truly dynamic S-box method for symmetric block ciphers.

## Motivation and contribution

Several dynamic S-Box solutions (Gangadari & Ahamed, 2015; Agarwal, Singh & Kilicman, 2018; Das, Zaman & Ghosh, 2013) have been proposed in earlier years, which rely on affine transformation having arithmetic irreducible polynomial (11B) with additive constant (63) to handle substitution operation in symmetric cryptosystems. The use of any known S-box transformation or any additive constant is not good way to create dynamic substitution, because, the known parameters always help the cracker in cryptanalysis. Thus, the predefined substitution with publically know S-Box is a noteworthy challenge with current symmetric cryptosystems (Katiyar, Jeyanthi & Roperties, 2016). S-boxes have significant role in providing confusion to block ciphers and should be created dynamically to increase cryptographic strengths (Hosseinkhani & Haj Seyyed Javadi, 2012; Pradeep & Bhattacharjya, 2013) as well as to resist differential and linear attacks significantly (Ara, Shah & Prabhakar, 2018). Several strong S-boxes have been designed by researchers (Zahid, Arshad & Ahmad, 2019; Ahmad, Doja & Beg, 2018; Hussain et al., 2018), but all these solutions are static (fixed) in nature and not optimally secure (Das, Zaman & Ghosh, 2013; Devi, Sharma & Rangra, 2015). Therefore, there is need of dynamic key dependent substitution method alike the proposed substitution method which should be free from publically known S-box transformation (irreducible polynomials, additive constants, static lookup table etc.) as compared to the others. The proposed substitution method presents its contribution in enhancing security of symmetric cryptosystems by generating all the values of S-box at the time of execution from the secret key. It uses 128-bits secret key and performs dynamic left circular shift, exclusive-OR and other simple permutations for generation of 256 values of S-box. In contrast to the existing solutions, the proposed S-box method always generates unique and dynamic S-box values each time during execution. Moreover, the proposed S-box method is also optimally strong in randomness and cryptographic properties in comparison with others. Additionally, the proposed method is useful in achieving the basic goals of security i.e. confidentiality, authenticity, integrity and non-repudiation. As these goals have been stated in various studies (Musliyana, Arif & Munadi, 2015; Agrawal, 2012; O’Melia & Elbirt, 2010; Nejad, Sabah & Jam, 2014; Fahmy et al., 2005).

## Related work

### Existing substitution methods

To upgrade the static S-box structure of AES with dynamic properties, several efforts have been made in earlier years. In 2013, Das et al. proposed a key dependent S-box method (Das, Zaman & Ghosh, 2013) with constant and additive natured irreducible polynomials for generating of different S-boxes. In Kazlauskas, Vaicekauskas & Smaliukas (2015), designed S-boxes by performing different operations on round key. Initially, static S-box of AES was used in 1^st^ round to generate various S-boxes which retain the resistance against differential and linear cryptanalysis. However, the generated S-boxes were not only based on static S-box of AES but the avalanche properties of these generated s-boxes were also not optimum. The 1^st^ byte of round key (generated through key scheduling) with static s-box of AES was used in Nejad, Sabah & Jam (2014) to construct AES based key dependent substitution function, which was lacked in several aspects such as execution time, dynamic properties and resistance against modern attacks.

The two parameters (GNU-C and ISO-C) were used to generate S-box for symmetric cryptosystems in which instead of inverse S-box, a new transformation (shift row transformation) was introduced (Fahmy et al., 2005). The list of 30 irreducible polynomials with affine values ranging from 0 to 255 were used by Agarwal, Singh & Kilicman (2018) to generate 256 key dependent S-boxes. But the generated s-boxes were not effective in security as the both strict avalanche and bit independence criteria of S-boxes were not evaluated. In Sahoo, Kole & Rahaman (2012), utlized affine transformation to create static S-boxes. Time complexity was reduced by using affine values differently without the considerations of security parameters. Whereas, security may not compromised in cryptography. A round-key dependent S-box method was proposed in Partheeban & Kavitha (2018) which contains high non-linearity but this scheme is also based on static S-box of AES to generate new S-boxes. Similarly, the multi-operation S-box construction strategy as used in Desai & Nadaf (2012) and Anees & Chen (2019) is also dependent on the static S-box of AES. In Abd-ElGhafar et al. (2009), suggested the use of stream cipher (i.e. RC4) to generate key dependent S-box. Affine transformation was implemented using RC4 to generate final RC4 based S-box. The RC4 based S-box was used for performing substitution instead of static S-box by considering two keys (one for encryption and other for generating of S-box).

In Waqas et al. (2015), altered the affine matrix for creation of 46 S-boxes. From a list of 255 affine matrices, 190 matrices were invertible. These invertible affine matrices were used for S-boxes generation which were also static in nature. In Singh & Singh (2019), Harpreet and Paramvir created key-dependent S-box and also proposed a new key scheduling algorithm by performing different operations (e. XOR, left rotation, nibble swap and SHA256) on 128 bits of key. Their strategy to create dependency between key and S-box was also static. The dynamic S-boxes construction approach (Manjula & Mohan, 2017), in which the static S-box was just left rotated according to the resultant values of 16 bytes of round key after performing exclusive-OR operation. The pseudo random numbers were used in Maram & Gnanasekar (2016), Alabaichi & Salih (2015) and Maram & Gnanasekar (2018) to generate dynamic S-box values, however, these approaches were not effective in creating dynamicity, strict avalanche properties etc. as compare to the proposed S-box scheme. Moreover, the key dependent S-box solutions as discussed in Juremi et al. (2012) and Wenceslao (2015) were also lacked in computational efficiency, dynamicity, avalanche criteria.

The in-depth literature analysis reveals that some S-box methods only focus on improving computational efficiency but these are deficient in dynamicity and randomness (Wenceslao, 2015; Sahoo, Kole & Rahaman, 2012). Moreover few methods have not been tested well (Hosseinkhani & Haj Seyyed Javadi, 2012; Agarwal, Singh & Kilicman, 2018; Biham & Shamir, 1991; Cusick & Stanica, 2017) and all these are not seems to be effective in achieving of optimal dynamicity and avalanche properties in contrast with proposed S-box scheme. Most of existing S-box schemes are static in nature thereby, these are not effective in generating of dynamic S-boxes. Therefore, there is need to design a dynamic S-box solution to improve strict avalanche criteria with optimal dynamicity, randomness and other common cryptographic properties.

### Structure and properties of AES based S-box

The S-box transformation or byte substitution is a non-linear operation which is performed independently on each byte. Therefore, the AES based S-box is invertible and can be constructed by composing two transformations (Gangadari & Ahamed, 2015).

By taking multiplicative inverse in GF(2^8^), where(x′)^−1^ as:

}{}x = {\left( {{x}^{\prime}} \right)^{ - 1}} = \left\{ {\matrix{ {{{\left( {{x}^{\prime}} \right)}^{ - 1}}\; \; \; {x}^{\prime} \ne 0} \cr {0\; \; \; \; \; \; {x}^{\prime} = 0} \cr } } \right.

By applying of affine transformation over GF(2) as:

\left[ {\matrix{ {\mathop S\nolimits_7 } \cr {\mathop S\nolimits_6 } \cr {\mathop S\nolimits_5 } \cr {\mathop S\nolimits_4 } \cr {\mathop S\nolimits_3 } \cr {\mathop S\nolimits_2 } \cr {\mathop S\nolimits_1 } \cr {\mathop S\nolimits_0 } \cr } } \right] = \left[ {\matrix{ 1 & 1 & 1 & 1 & 1 & 0 & 0 & 0 \cr 0 & 1 & 1 & 1 & 1 & 1 & 0 & 0 \cr 0 & 0 & 1 & 1 & 1 & 1 & 1 & 0 \cr 0 & 0 & 0 & 1 & 1 & 1 & 1 & 1 \cr 1 & 0 & 0 & 0 & 1 & 1 & 1 & 1 \cr 1 & 1 & 0 & 0 & 0 & 1 & 1 & 1 \cr 1 & 1 & 1 & 0 & 0 & 0 & 1 & 1 \cr 1 & 1 & 1 & 1 & 0 & 0 & 0 & 1 \cr } } \right]\left[ {\matrix{ {\mathop C\nolimits_7 } \cr {\mathop C\nolimits_6 } \cr {\mathop C\nolimits_5 } \cr {\mathop C\nolimits_4 } \cr {\mathop C\nolimits_3 } \cr {\mathop C\nolimits_2 } \cr {\mathop C\nolimits_1 } \cr {\mathop C\nolimits_0 } \cr } } \right] \oplus \left[ {\matrix{ 0 \cr 1 \cr 1 \cr 0 \cr 0 \cr 0 \cr 1 \cr 1 \cr } } \right]and

}{}b\left( x \right) = a\left( x \right)\left( {{x^4} + {x^3} + {x^2} + x + 1} \right) + \left( {{x^6} + {x^5} + x + 1} \right)mod\left( {{x^8} + 1} \right)

Where, *a(x)* and *b(x)* are algebraic expression. The design of AES based S-box is algebraic in nature having algebraic properties due to which algebraic attacks are most probably applicable.

Thus, algebraically, it is practically insignificant to construct a fully secure S-box due to bi-jective properties such as:

*(X ⊕ Y = Y ⊕ X)*

An S-box having order (m x n) is a mapping function }{}C = f\left( X \right), where\; f:{\left\{ {0,1} \right\}^m} \to {\left\{ {0,1} \right\}^n}, which is used to map m-bits input string X to n-bits output string C. it is lie under Boolean function and may be transformed to least sum (XOR⊕) of the products (AND •) as represented in Eq. (1). Therefore Boolean mapping: {0,1}^m^ → {0,1} set up an expression using Ӈ:∑^m→^ {0,1}taking binary sequence Ӈ(Ƶ0), Ӈ (Ƶ1),………, Ӈ (Ƶ2^n−1^) as lookup table of Ӈ. Some arrangement of Boolean expression: (−1) ^Ӈ(Ƶ1)^, (−1) ^Ӈ (Ƶ2)^,………….., (−1) ^Ӈ (Ƶ2n−1)^ is a subset of Ӈ. In case of ratio ½ of (0, 1) binary digits, the Boolean function provides a balanced transformation. However it can further be represented as

(1) \matrix{{(\beta 1 \ldots ,\beta {\rm{n}}) = } & {0 \oplus 1\cdot\beta 1 \oplus \cdot\cdot\cdot \oplus n\cdot\beta n \oplus 1,2.\beta 1\cdot\beta 2 \oplus \cdot\cdot\cdot\cdot\cdot\cdot \oplus n - 1,}  \cr {} & {n\cdot\beta n - 1\cdot\beta n \oplus \cdot\cdot\cdot \oplus 1,2...,n\beta 1\cdot\beta 2...\beta n.}  \cr }

The vector denoted with βn is the member of GF: }{}S{`_{box}} *= M •* }{}{S_{box}}^{ - 1}\;*+ C* and retains linear (one-to-one) relationship between βn and {0, 2^n^ – 1}.

There are two different Boolean functions, denoted with (*A* and *B* ), where *ᵴ ∈ A* and ƫ *∈ B*. The affine function’s set (F) and distance (D) will have non-linearity (}{}{\rm {\open N}}) as: }{}{\rm \; }{\rm {\open N}} = }{}{\rm Mi}{{\rm n}_{{\rm B} \in {\rm \; \; }{F }}}. Here F is set of affine functions upon *£*^*ᵴ*^ and ɭ*_j_* is the linear function of X.

(2) }{}{\rm D}\left( {A,B} \right) = {2^{n - 1}} - \displaystyle{1 \over 2} \unknown \unknown,\unknown \unknown

(3) }{}{\rm {\open N}} = {2^n} - \displaystyle{1 \over 2}Ma{x_{j = 0,1, \ldots \ldots ,{2^{n - 1}}}}\left\{ {\left| {\unknown, {}_{j}} \right|} \right\}

let *A* is a function over X_n_ and *U,N ∈ X*_*n*_, the *A* satisfies the propagation properties of *t(Ҫ*_*t*_*)*. where if propagation properties are: ∀* U ∈ X*_*n*_ *:1≤ W(U) ≤ t*, then the standards Ҫ_1_ fulfills strict avalanche criteria(*SAC*) (Shoukat et al., 2020b). Let W(.) shows the hamming weight of vector, whose element is 1. The W(.) of *A, B* can be calculated as: }{}{\rm D}\left( {A,\; B} \right) = \mathop \sum \nolimits_{\forall n:A\left( n \right)! = B\left( \right)} \; and the scalar product \unknown U,N \unknown = \oplus _{j = 0}^{n - 1}{U_i},\; {\rm \; }{N_i} bearing correlation immunity over *U,N ∈ X*_*n*_ only if *A* is balanced. The *A* consists of correlation immunity order *t’(K1*_*t*_*)* over *∀ U ∈ X*_*n*_ *:1≤ W(U) ≤ t*.

The *p × q* S-box (*S*) is treated as regular if *∀ N∈ X*_*q*_*: |*}{}S_\; ^{ - 1}*(N)|=* }{}{2^{p - q}} and *p × p* natured *S* is regular at bijective characteristic from (*X*_*p*_ *→ X*_*p*_ ). EX-OR Table in contrast to *S* with dimensions *(2*^*p*^ *× 2*^*q*^*)* of matrix with many elements:

\forall U \in }{}{X_v}, \forall N \in }{}{X_q} : }{}{D_{UN}} = \left| {\left\{ {w \in {X_v}|\; S\left( w \right) \oplus \; S\left( {w + U} \right) = N} \right\}} \right|Then assume that in *EX-OR table* if *α* is biggest number with non-zero γ in first *row* of the table then the both *2*^*p*^ values present in the left side at the top of the table will be abandoned and differential attacking strength (Φ) of *S* will be as:

(4) \Phi \left( S \right) = \left( {1 - \displaystyle{\gamma \over {{2^p}}}} \right)\left( {1 - \displaystyle{\alpha \over {{2^p}}}} \right)

Highest value of *Φ(S)* is good to resist against differential attacks.

Ideally, a good S-Box has to satisfy cryptographic properties, which include strict avalanche criteria, correlation coefficient and nonlinearity. If any S-Box satisfies these properties then it is considered as cryptographically secure (Gangadari & Ahamed, 2015).Therefore by making S-boxes dynamic and dependent on secret key these properties should necessarily be satisfied.

## Proposed method

### Operations used in proposed method

The proposed substitution method comprises of some simple but cryptographically significant mathematical operations or functions. The proposed method is not alike AES based S-box or its other variants because it does not utilize affine polynomials for generating of S-box values. In the proposed substitution method, S-boxes are created dynamically from 128-bits of the secret key by performing some simple operations such as circular shift followed by XOR and nibble swap.

In mathematics, “a circular shift is the operation of rearranging the numbers in a tuple” (Oshiba, 1972). A circular shift is an exceptional type of cyclic permutation (Gove, 1963). Formally, a permutation σ is a circular shift of *n* entries in each tuple such that:

(5) \sigma \left( i \right) \equiv \left( {i + 1} \right)modulo\; n,\; for\; all\; entries\; i = 1, \ldots ,n

Exclusive OR is a logical operation that returns a true value as an output if and only if when both inputs are different i.e. one is true and other is false. Its symbol is as follows:

(6) }{}A\; XOR\; B\; is\; written\; as\; A \oplus B

In nibble swap, the term nibble originally means “half a byte” or “half an octet”. The terms 'byte' and 'nibble' almost always refers to either 8-bits or 4-bits respectively. In nibble swap operation, a byte is separated form middle, into the two nibbles and then both nibbles change their position with each other. Dynamic S-boxes are created by using proposed method which is entirely different from static S-box of AES, as, it is constructed through mathematical operations to avoid algebraic attacks. The mathematical structure of the proposed method including these operations has been discussed in “Mathematical structure and step by step procedure”.

### Mathematical structure and step by step procedure

**Figure 1:**
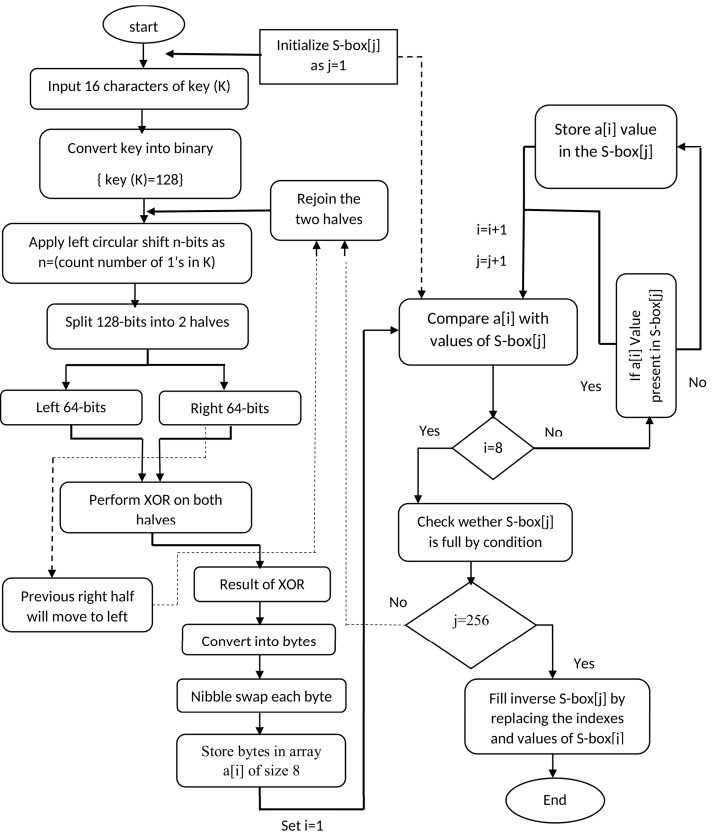
Working flow of proposed substitution method.

This section illustrates mathematical structure of proposed method with their working flow to generate dynamic S-box values. The step by step working of proposed method is presented in Fig. 1.

All the steps are performed to generate S-box; however, the procedure of generating of inverse S-box includes several steps such as:

- In 1^st^ step: 16 characters of 128 bits input encryption key (K) are converted into binary form. Then after counting 1s from 128-bits binary sequence, the left circular shift operation is applied on binary key according to the total number of ones. The circular shift permutation, has denoted with symbol “<<K_128_”. This permutation is dynamic which creates resistance against different attacks.
- In 2^nd^ step: 128-bits key is partitioned into left and right halves each having binary length of 64 bits. Both halves are denoted as LK_64_, RK_64_ and XOR operation is applied on two halves of key. After performing XOR operation, the resultant 64-bits are stored at right side, however the previous right-half with 64-bits are swapped to left side to be considered as new left-half with 64 bits.}{}{\left( {RK} \right)^{^\prime }} = L{K_{64}} \oplus R{K_{64}}}{}{\left( {LK} \right)^{\prime}} = R{K_{64}}
- In 3^rd^ step: right side 64-bits are converted into 8-bytes in hexadecimal form as: }{}hex{\left( {RK} \right)^{\prime}} = {k_1}{k_2}{k_3}{k_4}{k_5}{k_6}{k_7}{k_8}. Where }{}{k_1}{k_2}{k_3}{k_4}{k_5}{k_6}{k_7}{k_8} = {x_1}{y_1}{x_2}{y_2}{x_3}{y_3}{x_4}{y_4}{x_5}{y_5}{x_6}{y_6}{x_7}{y_7}{x_8}{y_8} After that, nibble swap is performed on each byte of right-half that is given as: }{}{x_1}{y_1}{x_2}{y_2}{x_3}{y_3}{x_4}{y_4}{x_5}{y_5}{x_6}{y_6}{x_7}{y_7}{x_8}{y_8} = {y_1}{x_1}{y_2}{x_2}{y_3}{x_3}{y_4}{x_4}{y_5}{x_5}{y_6}{x_6}{y_7}{x_7}{y_8}{x_8} Nibble swap helps to break patterns to create non-linear values of S-box. All the 8-bytes of right-half are stored in an array followed by a loop for placing these bytes into the S-box as: }{}{y_1}{x_1}{y_2}{x_2}{y_3}{x_3}{y_4}{x_4}{y_5}{x_5}{y_6}{x_6}{y_7}{x_7}{y_8}{x_8} = {S_1}{S_2}{S_3}{S_4}{S_5}{S_6}{S_7}{S_8}. After that, a conditional statement is used to ensure the uniqueness of S-box values to avoid any duplication.
- In step 4: after storing hex values of right-half in S-box, the right half is reconverted into binary (64-bits) as: }{}{\left( {RK} \right)_{64}} \leftarrow {y_1}{x_1}{y_2}{x_2}{y_3}{x_3}{y_4}{x_4}{y_5}{x_5}{y_6}{x_6}{y_7}{x_7}{y_8}{x_8}. After that, both left and right halves rejoin here to make 128-bits binary sequence, and then control moves back to the step-1as: }{}{K_{128}} = {(RK)_{64}} + {\left( {LK} \right)_{64}}. After that, all the operations are performed in previous order until the unique 256 values in hex form are stored in S-box. All the steps are controlled by the conditional statements under conditional loop which continue to run until the generation of dynamic S-box with 256 unique values.
- In step 5: a new loop is used to generate inverse S-box. For this purpose, indexes and values of generated S-box are swapped with each other to create inverse S-box.

As an example Tables 1 and 2 demonstrate the dynamic S-box and inverse S-box, which are created by applying the proposed substitution method on a key given in example 1.

**Table 1:** Key-dependent dynamic S-box from key given in example 1.

	0	1	2	3	4	5	6	7	8	9	a	b	c	d	e	f
0	a8	08	26	38	24	10	16	d1	9b	60	58	a2	61	aa	c6	82
1	0b	36	37	5b	b6	39	9c	9f	c9	97	7e	7c	9a	19	4f	b8
2	f6	8c	64	dc	40	88	76	90	14	d6	da	db	0d	0e	7b	3b
3	21	b2	73	7d	2b	ff	3c	f3	33	f9	22	17	e8	89	af	e4
4	5c	86	91	68	a3	e7	0f	cb	9d	b9	6e	2c	d4	e9	ae	84
5	1a	b5	fe	23	01	30	e6	1d	95	29	55	ce	6a	71	c4	96
6	6c	f5	5a	70	ef	2f	94	48	b0	4b	7f	77	2a	a1	69	ab
7	ca	a4	ad	f1	b7	dd	57	a7	51	12	87	6d	de	ee	d7	42
8	1f	a6	0c	92	11	15	fc	80	b1	45	28	4e	31	47	6f	ec
9	e2	34	c1	44	03	35	50	04	cc	0a	ea	8b	e0	1b	d9	ba
a	b4	3a	7a	c0	65	54	83	07	43	81	bc	1e	f8	32	b3	ed
b	99	3e	5d	2e	f4	e3	93	3d	a9	e1	18	a5	52	3f	66	05
c	c3	6b	20	d2	d8	d5	bd	25	63	56	fb	8a	4a	e5	8e	27
d	df	d3	4c	8f	bb	02	98	85	59	f2	be	ac	74	f7	bf	79
e	49	72	5f	9e	8d	4d	53	5e	cf	13	c2	eb	46	c7	fa	41
f	67	d0	fd	09	1c	a0	c8	2d	06	78	cd	f0	75	62	c5	00

**Table 2:** Key-dependent inverse S-box from key given in example 1.

	0	1	2	3	4	5	6	7	8	9	a	b	c	d	e	f
0	ff	54	d5	94	97	bf	f8	a7	01	f3	99	10	82	2c	2d	46
1	05	84	79	e9	28	85	06	3b	ba	1d	50	9d	f4	57	ab	80
2	c2	30	3a	53	04	c7	02	cf	8a	59	6c	34	4b	f7	b3	65
3	55	8c	ad	38	91	95	11	12	03	15	a1	2f	36	b7	b1	bd
4	24	ef	7f	a8	93	89	ec	8d	67	e0	cc	69	d2	e5	8b	1e
5	96	78	bc	e6	a5	5a	c9	76	0a	d8	62	13	40	b2	e7	e2
6	09	0c	fd	c8	22	a4	be	f0	43	6e	5c	c1	60	7b	4a	8e
7	63	5d	e1	32	dc	fc	26	6b	f9	df	a2	2e	1b	33	1a	6a
8	87	a9	0f	a6	4f	d7	41	7a	25	3d	cb	9b	21	e4	ce	d3
9	27	42	83	b6	66	58	5f	19	d6	b0	1c	08	16	48	e3	17
a	f5	6d	0b	44	71	bb	81	77	00	b8	0d	6f	db	72	4e	3e
b	68	88	31	ae	a0	51	14	74	1f	49	9f	d4	aa	c6	da	de
c	a3	92	ea	c0	5e	fe	0e	ed	f6	18	70	47	98	fa	5b	e8
d	f1	07	c3	d1	4c	c5	29	7e	c4	9e	2a	2b	23	75	7c	d0
e	9c	b9	90	b5	3f	cd	56	45	3c	4d	9a	eb	8f	af	7d	64
f	fb	73	d9	37	b4	61	20	dd	ac	39	ee	ca	86	f2	52	35

Example 1: key value (in hex): 7468617473206D79206B756E67206675

Moreover, S-box and inverse S-box generation algorithm is given in Table 3. Only one example of S-box and inverse S-box is given in this research paper. While the proposed method is capable of generating unlimited S-boxes and their inverse S-boxes as well, because proposed method is key dependent and a single bit change in key significantly results an entirely different S-box with unique values.

**Table 3:** S-box and inverse S-box generation algorithm.

**Dynamic** key**-dependent S-box and inverse S-box Generation Algorithm**
Input: binary sequence input by user, where K = 128 Output: S-box[j] and InvS-box[j], where j = 0,1,2……..,255 Initialization:t = 0 Step 1: n **←** count 1’s from K, perform nth time left circular shift. Step 2: divide K into two halves → R_64_, L_64_ Step 3: new R← R_64_ XOR L_64_, new L ← prev R_64_ Step 4: convert new R into bytes, i.e. K_1_, K_2_, K_3_, K_4_, K_5_, K_6_, K_7_ and K_8_ Step 5: perform nibble swap on each byte. Step 6: Store values in array a[i] For t = 0 to 255 for i =1 to 8 a[i] = K_i_ compare a[i] with values of S-box[j] for j = 0 to t If a[i] != S-box[j] S-box[j] = a[i] j = j + 1 t = t + 1 end if end for i = i + 1 end for rejoin the two halves goto step 1 end for Step 7: generate InvS-box[j] by using indices of S-box[j] End

## Results

This section illustrates the experimental results and findings of proposed S-box method. Proposed method has been evaluated with variety of measures such as nonlinearity, hamming distance, bit-independence, avalanche properties including differential and linear approximation analysis etc.

### Nonlinearity

Any good S-box should not have linear mapping from an input to an output because linear mapping weakens the cipher. Non-linearity with higher esteem makes the cipher more resistant to linear cryptanalysis (Zahid, Arshad & Ahmad, 2019; Partheeban & Kavitha, 2018). Non-linearity of S-boxes can be calculated as (Biham & Shamir, 1991; Cusick & Stanica, 2017):

(7) }{}NL = {2^{n - 1}} - \displaystyle{1 \over 2}\; \left( {\mathop {\max }\limits_{q \in {{\left\{ {0,1} \right\}}^n}} \left| {{W_f}\left( q \right)} \right|} \right)

Here, W_f_ (q) is Walsh-Hadamard spectrum and is measured as:

(8) }{}{W_f}\left( q \right) = \mathop \sum \limits_{p\epsilon {{\left\{ {0,1} \right\}}^n}} {\left( { - 1} \right)^{f\left( p \right) \oplus p.q}}

However, the q\; {\rm \epsilon }{\rm \; }{0,1}^n^. To validate non-linearity of proposed S-box method, several S-boxes were generated and their non-linearity was calculated. The non-linearity of proposed S-box method has been summarized in Table 4 in comparison with earlier dynamic natured S-box methods. The non-linearity values of proposed method (Min: 102, Max: 111 and Avg: 106.5) are significantly better than the non-linearity values of previous S-box methods (Kazlauskas, Smaliukas & Vaicekauskas, 2016; Hussain Alkhaldi, Hussain & Gondal, 2015; Hussain et al., 2011a; Hussain et al., 2011c). Thus, non-linearity is a good indicator for any s-box to resist linear attacks.

**Table 4:** Non-linearity comparison between proposed method and existing S-boxes.

S-box methods	Non-linearity		
	Minimum	Maximum	Average
(Mahmoud et al., 2013)	96	110	104.3
(Kazlauskas, Smaliukas & Vaicekauskas, 2016)	98	108	102.5
(Hussain Alkhaldi, Hussain & Gondal, 2015)	98	108	104
(Khan et al., 2012)	102	108	105.3
(Hussain et al., 2011a)	98	108	104
(Hussain et al., 2013)	100	108	104.8
(Siddiqui & Afsar, 2016)	104	106	105.3
(Hussain et al., 2011c)	94	104	99.5
Proposed method	102	111	106.5

### Linear approximation probability

Actually, the linear approximation probability was introduced in 1993 to break 8-rounds of DES (Matsui, 1994).The maximum value of imbalance of an event has been denoted with LP in which the parity of input bits selected by the mask A_u_ is equal to the parity of output bits selected by mask B_v_ as shown in Eq. (9).

(9) }{}LP = \mathop {\max }\limits_{{A_{u,}}{B_v} \ne 0} \left| {\displaystyle{{\# \left\{ {u \in U|u.{A_u} = S\left( u \right).{B_v}} \right\}} \over {{2^n}}} - \displaystyle{1 \over 2}} \right|

In Eq. (9), U represents the set of all possible inputs, A_u_ is input mask, B_v_ is output mask and 2^n^ is the number of elements with *n* = 8 (i.e. 2^8^ = 256). Different S-boxes were generated through proposed S-box method and their LP value was calculated by using formula as represented in Eq. (9). The LP results (Table 5) show that the maximum LP value of proposed substitution method is 0.109, which is better than maximum LP values of earlier S-box methods (Khan et al., 2012; Hussain et al., 2011b; Hussain et al., 2011a). Comparison shows that proposed substitution method retains significant linear approximation probability (LP) to resist linear attacks.

**Table 5:** LP comparison between proposed method and other S-boxes.

S-box methods	(Khan et al., 2012)	(Hussain et al., 2011b)	(Hussain et al., 2011c)	(Hussain et al., 2011a)	Proposed method
**Max LP**	0.140	0.1328	0.109	0.1328	0.109

### Differential approximation probability

In Biham & Shamir (1991) differential cryptanalysis of S-boxes was demonstrated by Biham and Shimar (Belazi, Rhouma & Belghith, 2015). The differential uniformity (Farwa et al., 2017) of any S-box can be measured through Eq. (10). According to it, an input differential ∆u_i_ should uniquely be mapped to an output differential ∆v_i_, to ensure uniform mapping.

(10) }{}DP\left( {{\rm \Delta}u \to {\rm \Delta v}} \right) = max\left[ {\displaystyle{{\# \left\{ {u \in U|S\left( u \right) \oplus S\left( {u \oplus {\rm \Delta}u} \right) = {\rm \Delta}v} \right\}} \over {{2^n}}}} \right]

Here U is the set of all possible input values, ∆u represents input differentials, ∆v shows the output differentials and 2^n^ represents the number of elements with *n* = 8 i.e. 2^n^ = 256. All the differential approximation values of proposed substitution method have been summarized in Table 6. From Table 6, it is clear that the maximum value is 10 which appears only for nine times in Table 6 and when the value 10 is divided by 256, the differential probability (DP) value becomes 0.03906. The comparison of DP value of proposed method with DP value of other S-boxes has been represented in Table 7. The overall analysis shows that the maximum DP value of proposed method is better than maximum DP values of earlier S-box methods (Hussain et al., 2011b; Jakimoski, 2001; Wang et al., 2009; Özkaynak & Yavuz, 2013) as shown in Table 7. Thus, the proposed substitution method is strong enough to resist differential attacks.

**Table 6:** Differential approximation table.

–	6	8	8	8	8	8	6	6	6	6	10	6	8	8	8	8
6	6	6	6	4	6	6	8	8	8	8	8	8	6	6	6	8
8	6	8	8	8	10	8	8	8	8	6	6	8	8	8	8	8
6	8	8	8	8	8	8	6	6	6	6	6	8	6	8	8	6
6	8	8	6	6	6	6	6	8	6	6	6	8	8	8	8	8
6	8	8	8	8	8	4	10	8	8	8	8	8	6	8	6	6
8	6	6	6	6	6	6	6	8	8	8	6	8	8	8	6	6
6	6	6	6	8	8	8	8	4	8	8	8	10	6	6	6	6
6	8	8	8	6	8	8	8	8	8	6	6	6	6	6	6	10
6	6	6	6	6	8	8	8	6	6	8	6	6	6	8	8	8
6	6	8	8	8	8	6	6	8	6	8	8	8	8	4	8	8
10	8	8	8	6	6	6	6	8	8	8	8	6	6	6	8	8
6	8	8	8	8	6	6	4	6	6	8	8	8	8	10	8	8
8	8	6	6	10	6	8	8	8	8	8	8	8	6	8	8	8
8	6	6	6	6	8	8	6	6	6	6	8	8	8	8	6	8
4	8	8	8	8	6	8	8	8	8	6	10	6	6	6	8	8

**Table 7:** Maximum DP comparison of proposed method with other S-boxes.

S-box method	(Khan et al., 2012)	(Hussain et al., 2011b)	(Jakimoski, 2001)	(Wang et al., 2009)	(Özkaynak & Yavuz, 2013)	(Çavuşoğlu et al., 2017)	Proposed method
Max DP	0.03906	0.0468	0.0468	0.0468	0.0468	0.03906	0.03906

### Avalanche effect

Avalanche effect is an important measure for cryptographic algorithms. For any cryptographic algorithm, the avalanche effect needs to be satisfied in such a way, changing of one binary bit in an input should result significant change in an output binary sequence generated by the cryptographic algorithm. In Eq. (11), the standard formula for measuring avalanche effect has been represented.

(11) }{}AE = \displaystyle{{Bits\; flipped\; in\; ciphertext} \over {Total\; bits\; of\; Ciphertext}} \times 100

**Figure 2:**
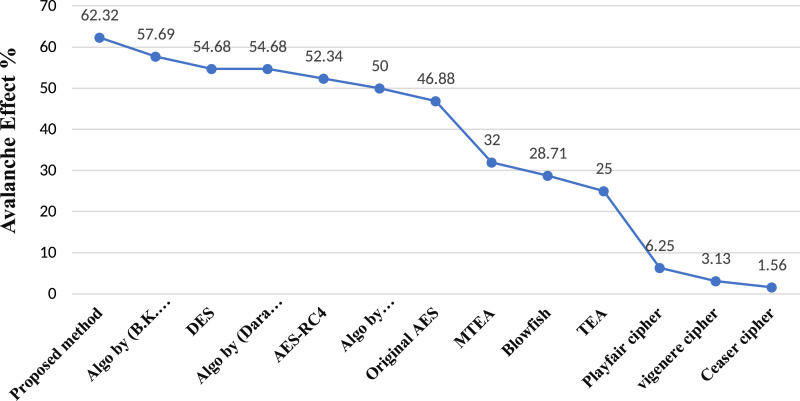
Avalanche comparison between proposed and existing methods.

The avalanche effect of proposed method has been tested several times through Eq. (11) and as result, the proposed method has shown average avalanche effect with 62.32% as depicted in Fig. 2. The earlier methods Maram & Gnanasekar (2016), Dara & Manochehri (2014), Dara & Manochehri (2013) and Kazlauskas & Kazlauskas (2009) retain 60% avalanche effect which is several time reduced than the avalanche effect (62.32%) of proposed method. When any cryptographic algorithm shows higher avalanche effect, it means the inside utilized mathematical functions and logic of that algorithm is cryptographically strong. According to Abd-ElGhafar et al. (2009) and Maram & Gnanasekar (2018), it is declared that, any cryptographic algorithm will be considered as secure against attacks as much as it will retain avalanche effect. Thus, the avalanche effect of proposed method is higher than others.

### Strict avalanche criteria

The generated cipher fulfills strict avalanche criteria (SAC), if the output changes with probability of ½ by alteration of 1-bit in an input binary sequence of any cryptographic algorithm (Peng & Jin, 2013). For calculation of SAC, 10,000 samples of plaintext were encrypted by using dynamic S-boxes produced by proposed substitution method. Results show that the proposed method clearly satisfies strict avalanche criteria. The dependence matrix for the SAC of proposed method is calculated as represented in Table 8. A comparison of minimum, maximum and average SAC values of the proposed substitution method with the SAC values of existing S-boxes have been summarized in Table 9. Thus, the proposed method also satisfies strict avalanche criteria significantly in comparison with others.

**Table 8:** Dependency matrix for SAC.

0.4902	0.5797	0.4992	0.5926	0.4902	0.4907	0.5264	0.4923
0.5372	0.4995	0.6232	0.4992	0.5215	0.4882	0.4995	0.4901
0.4955	0.4892	0.5627	0.5214	0.4901	0.5014	0.5827	0.5354
0.49	0.5399	0.4892	0.4901	0.4957	0.4926	0.5264	0.4902
0.4852	0.4902	0.4967	0.4889	0.4807	0.5128	0.4902	0.4905
0.4932	0.5178	0.5103	0.5062	0.4901	0.4909	0.5379	0.4867
0.5	0.4915	0.4902	0.4901	0.5124	0.4889	0.4997	0.5024
0.4915	0.4865	0.4917	0.5967	0.4905	0.6015	0.5843	0.4934

**Table 9:** Dependency matrix for SAC.

S-box methods	SAC		
	Min	Avg	Max
(Çavuşoğlu et al., 2017)	0.4218	0.5039	0.5937
(Jakimoski, 2001)	0.3761	0.5058	0.5975
(Wang et al., 2009)	0.4850	0.5072	0.5150
(Khan et al., 2012)	0.3906	0.5039	0.6250
(Özkaynak & Yavuz, 2013)	0.3906	0.4931	0.5703
(Hussain et al., 2011b)	0.3986	0.5032	0.5938
Proposed method	0.4807	0.5097	0.6315

### Correlation coefficient

It is considered as the significant aspect for the block ciphers security (Alabaichi & Salih, 2015). Correlation coefficient deals with dependency between input and output bits (Salih, Alabaichi & Tuama, 2020). It is a good source to know that how the two variables can effect each other. Correlation coefficient is also used to scale the degree of dependency of two individual variables on each other. Confusion effect can also be determined by the use of correlation coefficient over the block ciphers. The correlation coefficient value lies between (−1) and (1). The value (−1) shows that there is a decreasing linear relationship where the value (1) shows an increasing linear relationship. In case the value is “0”, then it means that both variables are independent (Mahmoud et al., 2013).

Equations (12)–(14) show the standard formula for correlation coefficient (r) of the data pairs (X_i_, Y_i_).

(12) }{}{r_{x,y}} = \displaystyle{{cov\left( {X,Y} \right)} \over {{\delta _x}{\delta _y}}}

Here,

(13) }{}cov\left( {X,Y} \right) = \displaystyle{1 \over {\left( {n - 1} \right)}}\mathop \sum \limits_{i = 1}^n \left( {{X_i} - {\mu _x}} \right)\left( {{Y_i} - {\mu _y}} \right)

And

(14) }{}{\delta _x}{\delta _y} = \sqrt {\displaystyle{{\mathop \sum \nolimits_{i = 1}^n {{\left( {{X_i} - {\mu _x}} \right)}^2}\mathop \sum \nolimits_{i = 1}^n {{\left( {{Y_i} - {\mu _y}} \right)}^2}} \over {{{\left( {n - 1} \right)}^2}}}}

**Figure 3:**
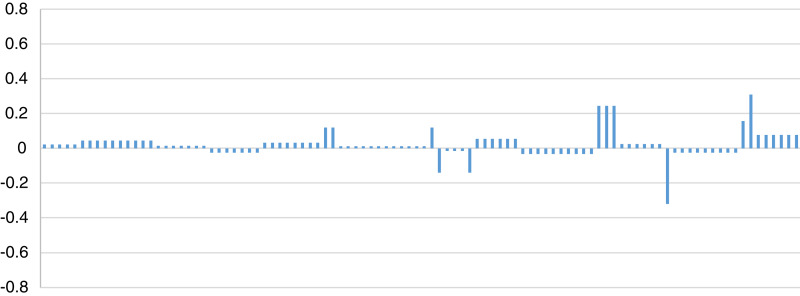
Correlation coefficient of the proposed method.

In Eq. (14), the X_i_ represents the values of the plaintext, Y_i_ represents the corresponding values of the cipher text and (μ_x_, μ_y_) are their mean values respectively. The correlation coefficient of 100 different sequences related to proposed method was calculated through standard correlation coefficient formula and resultant values have been depicted in Fig. 3. Average correlation coefficient (0.025) of the proposed method is close to zero which clearly invokes that both plaintext and the cipher-text are independent of each other without having linear relationship.

### Bit independence criterion

A function (*f*) justifies bit-independence criterion (BIC) for input (*x*) and output (*y, z*), in such a way, if the input bit (*x*) is inverted then the output bits (*y, z*) should change independently (Webster & Tavares, 1986). Correlation must be calculated to measure the relationship between avalanche variable sets (Çavuşoğlu et al., 2017). The non-linearity based bit-independence-criterion (BIC-NL) of the proposed S-box method lies among this range (Min: 98, Max: 108, Average: 103.392) as summarized in Table 10. Moreover, the comparison of BIC-NL results of proposed method with existing S-box methods has been shown in Table 11. Thus, the BIC-NL based results show that the proposed S-box method significantly justifies non-linearity based bit-independence-criterion (BIC-NL).

**Table 10:** BIC-non-linearity matrix for proposed substitution method.

–	100	104	98	106	102	108	104
100	–	100	104	102	106	102	108
106	98	–	100	108	100	104	98
102	104	108	–	102	104	106	106
98	100	98	102	–	102	104	100
104	102	106	108	106	–	102	102
102	104	108	106	108	106	–	100
108	108	102	100	104	102	108	–

**Table 11:** Comparison of BIC-non-linearity between the proposed method and other S-boxes.

S-box method	(Çavuşoğlu et al., 2017)	(Hussain Alkhaldi, Hussain & Gondal, 2015)	(Hussain et al., 2011c)	(Chen, Chen & Liao, 2007)	(Khan et al., 2012)	Proposed method
**BIC-NL**	103.3	102.9	101.7	103.1	100.3	103.3

### Hamming distance

It is a way of measuring dissimilarity between two equal strings by counting the all positions at which corresponding digits or characters of given strings are different. The different input sample texts as available in Maram & Gnanasekar (2018) have been used to measure the hamming distance of proposed method. Hamming distance values of the proposed method have been compared with the values of AES based static S-box including the previously published S-box method as discussed in Maram & Gnanasekar (2018). All the hamming distance related results of proposed method with existing S-box methods have been summarized in Table 12. The overall hamming distance analysis shows that the proposed method has higher values of hamming distances in comparison with others. Higher hamming distance is a good trait for any cryptographic method to generate cryptographically strengthened cipher-text.

**Table 12:** Results for hamming distance of proposed and existing methods.

Sample Text (in bytes)	AES with static s-box	s-box by Maram & Gnanasekar (2018)	Proposed method
3132333435214023242531323334355e262a282932333 43524255e262a39303938375e25242332315e262a2423 4021223b2e2c2f3f2b5f293938373635333440	65	50	68
616161616161616161616161616161616161616161616 1616161616161616161616161616161616161616161616 1616161616161616161616161616161616161	60	49	58
32333423206a732062204d746d62667232333523206a 732062204d746d62667232333423206a732062204d746 d62667332333423206a732062204d746d62667220	63	50	65
2054686b7320697320474d5220496e73742e206f662054 6563682e20497420697320696e2052616a616d2035333 2313239205372696b616b756c616d2044697374	70	48	76
4141414141424242424261616161613131313131434343 434325252525252a2a2a2a2a28282828283e3e3e3e3e3c3c 3c3c3c3a3a3a3a3a22222222222d2d2d2d20	65	49	66
317e7e7e7e7e7e7e7e7e7e7e7e7e7e7e7e7e7e7e7e7e7e7e7e7e7e5f5f5f5f5f5f5f5f5f5f5f5f5f5f5f5f5f5f205f5f5f5f5f20606060	61	51	68
3132333435363738396162636465666768696a6b313131313 13131313131313132323232323232327a7a7a7a7a7a7a7a79 79797979797979792340215e262a25	63	47	73

### Balanced output

It means the cipher-text generated by any cryptographic method should have equal probability of both 0’s and 1’s. The balanced output test was performed on different ciphers, which were generated through proposed method. The balanced output results have been shown in Table 13, which clearly show that the cipher generated by the proposed method holds 0’s and 1’s with almost equal probability. A cipher having balanced output is considered to be a stronger cipher than others in resisting of linear attacks (Maram & Gnanasekar, 2016).

**Table 13:** Results for balanced output of proposed substitution method.

Sample text (in bytes)	Proposed method	
	0’s	1’s
3132333435214023242531323334355e262a28293233343524255e262a33 03938375e25242332315e262a24234021223b2e2c2f3f2b5f293938373633	265	248
616161616161616161616161616161616161616161616161616161616161616161616161616161616161616161616161616161616161616161616161	256	256
32333423206a732062204d746d62667232333523206a732062204d746d62667232333423206a732062204d746d62667332333423206a732062204d746d62667220	253	259
2054686b7320697320474d5220496e73742e206f6620546563682e20497420697320696e2052616a616d20353332313239205372696b616b756c616d2044697374	260	253
414141414142424242426161616161313131313143434343432525252525 2a2a2a2a2a28282828283e3e3e3e3e3c3c3c3c3c3a3a3a3a3a22222222222d2d2d2d20	244	268
317e7e7e7e7e7e7e7e7e7e7e7e7e7e7e7e7e7e7e7e7e7e7e7e7e7e5f5f5f5f5 f5f5f5f5f5f5f5f5f5f5f5f5f5f205f5f5f5f5f2060606060606060	253	259

### Difference percentage

This is a very important factor to analyze the strength of the S-boxes. This test analyzes that how many values are rearranged at a different position from previously generated S-box, when only a single bit of the key is altered. Another important property of this test is that it increases the avalanche effect to enhance its security. For apply this test, 1,000 S-boxes were generated by using 1,000 different keys and then again 1,000 more s-boxes were generated by changing 1-bit of each key out of 1,000 different keys. Thus all newly generated S-boxes were compared with previous S-boxes. It was found that the proposed method is capable of generating new S-box completely with unique values by changing just single bit of input key. As an example the S-box generated by the proposed method with this encryption key: 7468617473206D79206B756E67206675 is shown in Table 1. However, after changing only 1-bit in given encryption key, the proposed method generates totally different and unique S-box as represented in Table 14. Thus, the comparison of Table 1 and Table 14 shows that, the proposed method completely fulfills the criteria of difference percentage in generating of new S-box with different values even after changing of 1-bit in an encryption key.

**Table 14:** New differently arranged S-box by changing 1-bit in key.

	0	1	2	3	4	5	6	7	8	9	a	b	c	d	e	f
0	7c	04	13	14	1a	00	0b	30	e6	41	a4	18	ea	b9	94	10
1	78	f1	d5	fc	83	a9	4f	1c	8d	87	cd	08	6e	54	0e	47
2	bb	74	f8	38	0f	45	86	f0	55	62	e1	85	11	50	cb	d0
3	05	8b	63	f2	44	79	5a	a8	71	ec	9f	82	ca	7f	4a	e7
4	52	22	9e	3a	cc	99	4c	01	75	3c	2a	8e	21	ba	d8	58
5	95	ff	53	90	28	32	0d	c4	96	81	20	68	ce	80	40	da
6	09	b1	fd	4d	66	c5	ef	dd	db	73	1e	d4	57	69	b0	a6
7	2e	c0	37	9c	17	e5	35	a2	cf	98	33	8a	0a	49	d1	c3
8	5f	aa	b8	8f	ed	89	b2	3d	af	de	dc	72	d3	fe	f4	7d
9	d2	7e	6c	2b	07	70	f6	93	29	6a	3f	b4	5d	a3	ad	88
a	0c	5c	b5	a1	bf	bc	67	d9	25	19	c9	15	92	e0	f3	8c
b	64	2c	56	be	1f	46	4b	ab	5b	fa	4e	42	a7	fb	31	77
c	9b	2f	ac	f5	3e	9a	12	61	f9	02	3b	48	ee	e2	9d	b7
d	d6	36	6f	6b	5e	1b	1d	06	eb	e9	e4	d7	34	27	26	39
e	c7	43	97	16	df	c8	7a	84	b6	59	65	91	bd	51	24	c6
f	7b	60	e8	c2	a5	f7	c1	ae	03	e3	2d	23	76	b3	a0	6d

### NIST statistical tests

National Institute of Standard and Technology (NIST) recommends Statistical Testing Suit (STS) with several standard tests to verify the randomness and statistical properties of the cipher-text generated through a newly developed cryptosystem. All those standard tests which are based on verifying the probability (p) of occurring 0 and 1 in any given binary sequence require that *p*-value should remain in between (0.01–1.00). While performing any NIST standard test, once the *p*-values lie from 0.01 to 1.00 then it means the test is successful to fulfill the required level of randomness as set by the NIST. In case, if the cipher-text generated through any cryptosystem fails to fulfill the criteria of *p*-values (}{}{\rm}0.01 < p - value \le 1.00) then it is considered as un-successful (fail). Therefore, the cipher-text generated by the proposed method has been tested with several statistical tests (“Frequency (Mono-bit) Test”, “Frequency Test within a Block”, “Runs test”, “Test for Longest-Run-of-Ones in a Block”, “Non-overlapping Template Matching Test” and “Overlapping Template Matching Test”) recommended by NIST to verify its randomness properties.

#### Frequency (mono-bit) test

This test is performed to know, that whether in any sequence the number of 0’s and 1’s are equal, as it is expected for a random sequence. Its standard formula is given in Eq. (15):

(15) }{}p - value = erfc\displaystyle{{{S_{obs}}} \over {\sqrt 2 }}

#### Frequency test within a block

It is performed to verify that either frequency of 0’s or 1’s is ½ in each block or not. Its formula is represented in Eq. (16):

(16) }{}p - value = igamc\left( {\displaystyle{N \over 2},\displaystyle{{{\chi ^2}\left( {obs} \right)} \over 2}} \right)

#### Runs test

This test verifies, whether the runs of 1’s and 0’s of different lengths are up to the acceptable range as set by NIST for any random sequence. The runs-test formula is available in Eq. (17):

(17) }{}p - value = erfc\left[ {\displaystyle{{\left| {{V_n}\left( {obs} \right) - 2n\pi \left( {1 - \pi } \right)} \right|} \over {\root 2 \of {2n\pi } \left( {1 - \pi } \right)}}} \right]

#### Test for longest-run-of-ones in a block

The main objective of this test is to verify, that the length of the longest run of 1’s is consistent in limits of the tested sequence with length of the expected longest run of 1’s in any random sequence. Its formula is shown in Eq. (18):

(18) }{}p - value = igamc\left[ {\displaystyle{K \over 2},\displaystyle{{{\chi ^2}\left( {obs} \right)} \over 2}} \right]

#### Non-overlapping template matching test

This test is performed to find that how many times a specific pattern of bits occurs through-out in the given testing sequence. Its standard formula is represented in Eq. (19):

(19) }{}p - value = igamc\left[ {\displaystyle{N \over 2},\displaystyle{{{\chi ^2}\left( {obs} \right)} \over 2}} \right]

#### Overlapping template matching test

This test is performed to search for the occurrences of an m-bit pattern within the sequence to be tested. Its formula is given in Eq. (20):

(20) }{}p - value = igamc\left[ {\displaystyle{n \over 2},\displaystyle{{{\chi ^2}\left( {obs} \right)} \over 2}} \right]

The different output sequences (strings) of proposed method have been evaluated through these discussed standard tests and the results have been summarized in Table 15. The p-value based results clearly invoke that, the outcomes achieved through the proposed method have passed out the randomness criteria as set by NIST. Thus, the proposed method retains good randomness properties as it is expected from any strong cryptographic method.

**Table 15:** Results of NIST test suite for proposed method.

Test ID	Test name	Proposed method *p*-values	Result
1	frequency	0.52137	Pass
2	Block frequency test	0.49166	Pass
3	Runs test	0.52343	Pass
4	Longest runs of 1’s	0.51692	Pass
5	Non-overlapping (m = 4, B = 0001)	0.43175	Pass
6	Non-overlapping (m = 4, B = 0011)	0.46749	Pass
7	Non-overlapping (m = 4, B = 0111)	0.44740	Pass
8	Non-overlapping (m = 4, B = 1000)	0.46273	Pass
9	Non-overlapping (m = 4, B = 1100)	0.45967	Pass
10	Non-overlapping (m = 4, B = 1110)	0.48632	Pass
11	Overlapping templates	0.565576	Pass

### Side-channel attack

**Figure 4:**
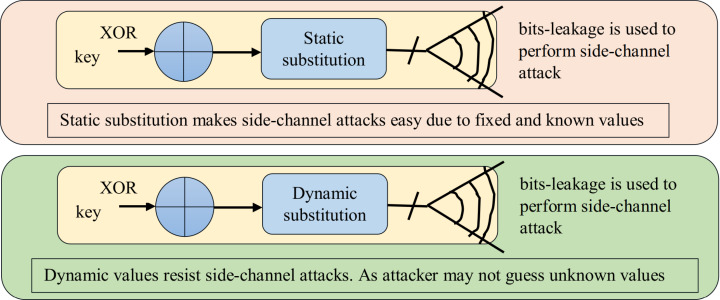
Bits-leakage side-channel attack scenario for static and dynamic substitutions.

The S-boxes generated by proposed method are fully dynamic and cryptographically strong as it has been justified through several experimentations. The advantage of dynamic substitution over static s-box is that, the dynamic s-boxes can resist linear, differential as well as side channel attacks significantly as witnessed in Suana (2018). Static s-boxes help the cracker to launch side channel attacks (Kazlauskas & Kazlauskas, 2009) due to the leakage of information acquires from static natured secret parameters. Moreover, static substitution, allows an attacker to exploit the leakage and to extract the secret information e.g. key (Chen, Chen & Liao, 2007). As the proposed substitution method does not have any static S-box, instead the S-boxes are created dynamically at time of execution of the algorithm. So the secret and dynamic s-box values of proposed method is not known to cracker, which saves the proposed method from side channel attacks. Although AES and other known algorithms with static substitution are resistant to many attacks but still side channel attacks are possible with AES based static S-boxes as discussed in Sasdrich et al. (2015) and Carlet et al. (2020). Whereas in proposed substitution method every time S-box generates different and unique values according to different keys and attackers will not be able to guess the S-box values. The dynamic S-boxes generated by proposed substitution method are more resistant against attacks, as the dynamic values help to resist side-channel attacks (Sasdrich et al., 2015). Bits leakage side-channel attack scenario for static and dynamic substitutions are shown in Fig. 4, this scenario is presented in Carlet et al. (2020) also. Figure 4 clearly shows that dynamic values help to resist side-channel attacks.

## Discussion

To analyze the proposed substitution method, different keys were randomly selected to generate different S-boxes by using proposed method. Only one of the generated S-boxes is represented in Table 1 as an example. The use of dynamic S-box is good to achieve effective confusion in symmetric block ciphers to resist modern cryptanalysis (Shoukat et al., 2020b). A good and cryptographically strong S-box should also have higher nonlinearity. The proposed method contains average nonlinearity (106.5) which is better than several existing S-box methods (Mahmoud et al., 2013; Kazlauskas, Smaliukas & Vaicekauskas, 2016; Hussain Alkhaldi, Hussain & Gondal, 2015; Khan et al., 2012; Hussain et al., 2011a; Hussain et al., 2013; Siddiqui & Afsar, 2016; Hussain et al., 2011c). Any S-box method enriched with higher nonlinearity alike proposed method is considered as strong to resist linear cryptanalysis. The proposed method has shown good linear and differential approximation probabilities (0.109, 0.03906) respectively which are quite significant to resist linear and differential attacks. Moreover, the proposed method contains average avalanche effect (62.32%) which is better than others (Maram & Gnanasekar, 2016; Dara & Manochehri, 2014; Dara & Manochehri, 2013; Kazlauskas & Kazlauskas, 2009) as depicted in Fig. 2. Similarly the strict avalanche criteria (average: 0.5091) of proposed method is also good to be declared it as a strong cryptographic method. The hamming distance of proposed method is also better than the static S-box of AES and a previously published S-box method as discussed in Maram & Gnanasekar (2018). The proposed method is capable to generate balanced output (Table 13) to resist modern attacks. Moreover, the proposed method has passed out several NIST based standard statistical tests (Table 15) through which all p-values lie in an acceptable range (}{}{\rm}0.01 < p - value \le 1.00) to satisfy randomness properties. The security strength of symmetric block ciphers is highly dependent on dynamicity and randomness properties (Shoukat, Bakar & Ibrahim, 2014). Thus, the proposed method includes good dynamicity and randomness. In future, the design of symmetric block ciphers needs to be evolved with dynamic features such as dynamic S-box, dynamic data blocks and selection of dynamic operations for each data block (Shoukat et al., 2020a).

## Conclusion

This research concludes that the key-dependent dynamic substitution method developed for symmetric cryptosystems is better than existing substitution methods in terms of generating dynamic S-box with all 256 values of S-box at different positions. The proposed dynamic substitution method (S-box) is capable to generate highly non-linear S-boxes as compare to others. The proposed method not only satisfies strict avalanche criteria but it also retains 62.32% average avalanche effect rather to others. Therefore, the proposed dynamic substitution method is strong enough to prevent symmetric block ciphers from linear and differential attacks as it has shown good results in term of non-linearity, linear and differential approximation probabilities. In showing of hamming distance, the proposed method is also better than static natured AES typed S-box. Moreover, the proposed method was also tested for Bit Independence Criteria, balanced output, correlation coefficient and as a result it has been found significant in comparison with existing S-box schemes. Furthermore, the proposed method has successfully cleared the standard security and randomness tests as recommended by NIST to validate its randomness properties. Thus, the overall experimentations shows that, the proposed substitution method is highly sensitive in generating of dynamic S-boxes with unique values and is significantly effective in improving the security of symmetric cryptosystems

## Additional Information and Declarations

### Data Availability

The following information was supplied regarding data availability: The data is available at GitHub: https://github.com/aisha-ejaz1819/uni-project1819.git.
